# Differential Gene Expression in *Rhododendron fortunei* Roots Colonized by an Ericoid Mycorrhizal Fungus and Increased Nitrogen Absorption and Plant Growth

**DOI:** 10.3389/fpls.2016.01594

**Published:** 2016-10-25

**Authors:** Xiangying Wei, Jianjun Chen, Chunying Zhang, Dongming Pan

**Affiliations:** ^1^College of Horticulture, Fujian Agriculture and Forestry UniversityFuzhou, China; ^2^Department of Environmental Horticulture and Mid-Florida Research and Education Center, Institute of Food and Agricultural Sciences, University of Florida, ApopkaFL, USA; ^3^Shanghai Academy of Landscape Architecture Science and PlanningShanghai, China

**Keywords:** Ericaceae, ericoid mycorrhiza, nitrate uptake, *Rhododendron fortunei*, symbiosis, transcriptome analysis

## Abstract

Ericoid mycorrhizal (ERM) fungi are specifically symbiotic with plants in the family Ericaceae. Little is known thus far about their symbiotic establishment and subsequent nitrogen (N) uptake at the molecular level. The present study devised a system for establishing a symbiotic relationship between *Rhododendron fortunei* Lindl. and an ERM fungus (*Oidiodendron maius* var. maius strain Om19), quantified seedling growth and N uptake, and compared transcriptome profiling between colonized and uncolonized roots using RNA-Seq. The Om19 colonization induced 16,892 genes that were differentially expressed in plant roots, of which 14,364 were upregulated and 2,528 were downregulated. These genes included those homologous to ATP-binding cassette transporters, calcium/calmodulin-dependent kinases, and symbiosis receptor-like kinases. N metabolism was particularly active in Om19-colonized roots, and 51 genes were upregulated, such as nitrate transporters, nitrate reductase, nitrite reductase, ammonium transporters, glutamine synthetase, and glutamate synthase. Transcriptome analysis also identified a series of genes involving endocytosis, Fc-gamma R-mediated phagocytosis, glycerophospholipid metabolism, and Gonadotropin-releasing hormone (GnRH) signal pathway that have not been reported previously. Their roles in the symbiosis require further investigation. The Om19 colonization significantly increased N uptake and seedling growth. Total N content and dry weight of colonized seedlings were 36.6 and 46.6% greater than control seedlings. This is the first transcriptome analysis of a species from the family Ericaceae colonized by an ERM fungus. The findings from this study will shed light on the mechanisms underlying symbiotic relationships of ericaceous species with ERM fungi and the symbiosis-resultant N uptake and plant growth.

## Introduction

Fungi play vital roles in many microbiological and ecological processes. The mutualistic association of plant roots with mycorrhizal fungi is the most widespread terrestrial symbiosis (Parniske, 2008). Host plants provide fungi with carbon compounds for growth and reproduction, while the fungi enhance host nutrient uptake and water absorption and improve disease resistance (Harrison, 2005). There are two types of mycorrhiza: ectomycorrhizas and endomycorrhizas, depending on whether the fungal hyphae colonize the root intercellular spaces or develop inside cells. Endomycorrhizas are further divided into ericoid, orchid, and arbuscular mycorrhizas (AM; Bonfante and Genre, 2010).

Endomycorrhizal colonization is a complex process, and current research has largely focused on AM fungi (Stracke et al., 2002; Akiyama et al., 2005). At the presymbiotic stage of colonization, plant roots produce and release carotenoid phytohormones, specifically strigolactones. Two GRAS-type transcription factors [named after the first three members: GIBBERELLIC-ACID INSENSITIVE (GAI), REPRESSOR of GAI (RGA) and SCARECROW (SCR)], *NSP1* and *NSP2* (nodulation-signaling pathway 1 and 2), which are both indispensable for nodulation in *legumes* (Kaló et al., 2005; Smit et al., 2005), have been implicated in the regulation of strigolactone biosynthesis. Strigolactones induce spore germination and hyphal growth and branching (Gutjahr and Parniske, 2013). Fungi produce mycorrhizal factors to induce calcium oscillations in root epidermal cells to activate plant symbiosis-related genes. Some of these identified genes include *DMI1 (*does not make infections), *DMI2*, and *DMI3. DMI1* encodes a predicted ion channel (Ané et al., 2004) which acts upstream of calcium spiking. *DMI2* encodes a symbiosis receptor-kinase (SymRK) or nodulation receptor kinase (NORK) that functions in the nod-factor perception/transduction system to initiate a signal cascade leading to nodulation (Endre et al., 2002). *DMI3* is a calcium/calmodulin-dependent kinase (CCaMK; Lévy et al., 2004; Mitra et al., 2004). These genes are important for both AM formation and root nodulation. AM fungi form special types of appressoria called hyphopodia. As a consequence of sequential chemical and mechanical stimulation, plant cells produce a pre-penetration apparatus (PPA). A fungal hypha that extends from the hyphopodium enters the PPA, which guides the fungus through root cells toward the cortex. At present, the signals triggering the formation of the PPA are unknown. Phenotypic analysis of *Medicago truncatula* symbiotic mutants shows that *SymRK/DMI2* are required for PPA induction and that *DMI3* is required for a subset of genes to be induced during PPA formation. A potential plant receptor for fungal chitin derivatives has been identified in *Parasponia andersonii* (Op den Camp et al., 2011). Silencing of this putative receptor, a LysM receptor kinase, which is closely related to the NFR5/NFP Nod factor receptor kinases from the legumes *Lotus* and *Medicago*, led to loss of nodulation and AM formation (Op den Camp et al., 2011).

Mycorrhizal symbiosis can significantly enhance plant roots in nutrient acquisition. Mycorrhiza-mediated phosphate uptake has been well documented (Karandashov and Bucher, 2005). Recent studies show that mycorrhizal colonization can also improve plant roots in N absorption (Govindarajulu et al., 2005; Tian et al., 2010). AM fungi access N and transport absorbed N via arginine from extra- to intra-radical mycelia; and the arginine is broken down through the urease cycle into ammonium for transport into the plant (Govindarajulu et al., 2005; Tian et al., 2010). Ammonium transporters (AMTs) have been identified to be responsible for uptake of ammonium at the periarbuscular membrane (Parniske, 2008). Plants also possess another set of transporters for uptake of nitrate (NO_3_^-^): NO_3_^-^ transporters (NRTs), which are encoded by two distinct gene families (*NRT1* and *NRT2*). The NRT1 family mainly regulates the low-affinity transport system (LATS), and the NRT2 family regulates high-affinity transport system (HATS; von Wittgenstein et al., 2014). Thus, roots symbiotically associated AM fungi have both mycorrhiza-mediated uptake and plant uptake pathways for acquisition of N (Bucking and Kafle, 2015).

Ericoid mycorrhizal (ERM) fungi are a distinctive type of endomycorrhiza, which forms symbiotic relationships specifically with roots of plants in the family Ericaceae, commonly known as the heath or heather family. The symbiosis allows host plants to adapt to a broad range of habitats, particularly acidic and infertile growing conditions (Cairney and Meharg, 2003). Until now, there is little information regarding the molecular basis of symbiosis between ERM fungi and plants from Ericaceae. Additionally, mechanisms regarding N uptake in plants of Ericaceae remain controversial. Cranberry (*Vaccinium macrocarpon* Ait.), a member of the family of Ericaceae, was reported to be unable to use NO_3_^-^ as a sole source of N in hydroponic culture (Rosen et al., 1990; Smith, 1993). This has been explained by a notion that nitrification was typically assumed to be negligible at soil pH below 5.5 (Paul and Clark, 1989), a characteristic of the sphagnum bog habitat native to cranberry and many other Ericaceae (Read and Perez-Moreno, 2003; Read et al., 2004). The adaptation to such soil conditions has resulted in the loss of the capacity to absorb NO_3_^-^. A recent report, however, showed that inoculation with the fungus *Rhizoscyphus ericae* increased the capacity of cranberry to absorb NO_3_^-^ (Kosola et al., 2007), and the authors believe that NO_3_^-^ may play a greater role in N nutrition of cranberry than previously thought and that high capacity for NO_3_^-^ utilization is the ancestral state for the Ericaceae. Yin et al. (2010) also found that ERM fungi significantly increased the ability of *R. fortunei* to absorb N, especially in the form of nitrate. Therefore, a better understanding of how ERM symbiosis enhances N uptake is not only biologically but also practically important as it could help improve production of some economically important horticultural crops, such as cranberry, blueberry, and rhododendron.

RNA-sequencing (RNA-Seq) has revolutionarily advanced sequence-based gene discovery (Wang et al., 2009). It provides a unique combination of transcriptome-wide coverage, sensitivity, and accuracy for a comprehensive view of gene expression changes for a specific developmental stage or physiological condition (Martin et al., 2013). RNA-Seq is rapidly becoming the method of choice for uncovering multiple facets of transcriptome to facilitate the biological applications including the investigation of plant–microbe interactions (Knief, 2014).

The present study was intended to explore the symbiotic relationships of ericaceous plants with ERM fungi and mechanisms underlying N acquisition using *Rhododendron fortunei* and *Oidiodendron maius* var. Maius strain Om19 as model organisms. A system to initiate their symbiosis was established, and subsequent N uptake and plant growth were examined. Using RNA-Seq, expression profiling of *R. fortunei* roots colonized by Om19 was analyzed against non-colonized control. A total of 16,892 differential-expressed genes (DEGs) were identified. Results from this study could shed light on molecular mechanisms governing the symbiotic establishment between ERM fungi and plants in the family Ericaceae and manifest how the symbiosis improves plant growth and N uptake.

## Materials and Methods

### Plant Material and Ericoid Mycorrhizal Fungus

Seeds of *R. fortunei* were rinsed in running tap water for 2 h and surface sterilized four times, 5 min each, using 25% (v v^-1^) solution of commercial bleach (8.25% NaOCl) followed by rinsing in sterile distilled water three times. The sterilized seeds were germinated on a half-strength Economou and Read medium (Economou and Read, 1984) supplemented with 1.5% (w v^-1^) sucrose and 0.7% (w v^-1^) agar with a pH of 5.2. The germination took place in a culture room under a 16-h photoperiod provided by cool-white fluorescent lamps at a photon flux density of 50 mmol m^-2^ s^-1^. Meanwhile, a peat-based substrate was formulated by mixing dry Klasmann peat (Geeste, Germany) with dry sand (washed with deionized water and dried) at 2 to 1 ratio based on volume. A modified Melin-Norkans (MMN) nutrient solution (Xiao and Berch, 1992) devoid of malt extract and glucose was prepared where the N source was replaced by Ca(NO_3_)_2_ to a final N concentration of 3.79 mM and pH was adjusted to 5.2. The peat-based substrate was moistened with the modified MMN solution at 3 to 2 ratio by volume, and its pH was tested to be to 5.2. The substrate was filled into 120 cylindrical vessels (400 mL) with 100 mL each, covered with caps, and autoclaved at 121°C for 30 min. Two months after seed germination, seedlings were transferred to 120 culture vessels containing the sterilized substrate, five seedlings per vessel.

*Oidiodendron maius* Om19 (Wei et al., 2016) was cultured on MMN agar medium (Molina and Palmer, 1982; Zhang et al., 2009). This strain was isolated from hair roots of *R. fortunei* grown in Huading Forest Park, Zhejiang Province, China. The sequence of this strain’s rDNA internal transcribed space was submitted to the NCBI database under the accession number KU382495. After 2 weeks of culture on MMN, mycelia of Om19 were collected using a sterile 5-mm diameter cork borer. After removing extra medium to a thickness of 1 mm, the 5-mm diameter disks were cut into half (a surface area of 9.8 mm^2^) were inoculated into the peat-based substrate next to each of the five *R. fortunei* seedlings. A total of 60 vessels were inoculated, while the remaining 60 vessels without inoculation were considered the control treatment. The inoculated and uninoculated treatments were designated as JZ and WJZ, respectively. To record plant growth, seedlings from randomly selected six vessels per treatment were collected weekly for 10 weeks. Roots were washed away of the substrate debris, the fresh weight of five seedlings in each vessel were recorded after blotted with paper towel. Means were calculated, and data were presented as mean ± SE.

### Microscopic Observation

After fresh weight recoding, roots were immediately fixed in formaldehyde-acetic acid-ethanol (FAA) for 24 h and heated at 90°C for 1 h in 10% KOH. The roots were rinsed in water, acidified with 1% HCl, and stained in a lactophenol-trypan blue (0.05% trypan blue in lactophenol) for 5 min at 90°C (Phillips and Hayman, 1970; Zhang et al., 2008). Stained roots were cleared with fresh lactophenol. The roots were examined under light microscopy for the presence of fungus inside root cells. Root colonization was quantitatively assessed using the method described by Biermann and Linderman (1981) as the percentage of root length with internal hyphal coils. Root samples collected on week 6 were also observed under scanning electronic microscope (SEM) using the method described by Bonfante-Fasolo and Gianinazzi-Pearson (1979). All specimens were coated with gold and platinum and examined using a SEM at 20 kV.

### Plant Growth and N Uptake

Based on the Om19 colonization results, a second experiment was carried out for determining biomass accumulation and tissue N content. The experiment was conducted in the same way as described above where 60 vessels were inoculated with Om19 as JZ treatment and the remaining 60 without inoculation as WJZ treatment. The experiment was arranged as a randomized complete block design with six blocks, and each treatment had 10 vessels per block. Plants were grown in the same conditions mentioned above without supplying any additional nutrient elements. After 6 weeks of inoculation, entire seedlings (shoots and roots) from both JZ and WJZ were harvested by carefully removing substrate from roots with running tap water. Seedlings from each block (50 seedlings) as a replicate were oven-dried at 80°C for at least 48 h, dry mass (50 seedlings) was measured. Tissues were analyzed for total N using CNS Auto-Analyzer (VarioMAX, Elementar Americas, Mt. Laurel, NJ, USA).

### RNA Extraction and RNA-Seq

A third experiment was carried out to prepare root materials for RNA extraction. The experiment was the same as the second experiment except the total number of culture vessels that were 300. Culture of Om19 was inoculated to 150 vessels (JZ), and the remaining 150 were control treatment without inoculation (WJZ). After 6 weeks of inoculation, roots were harvested by carefully removing substrate with running tap water and rinsed with sterile deionized water. After removing water with filter paper, roots harvested from JZ and WJZ treatments were, respectively, frozen in liquid N and stored at -80°C. Total RNA was extracted with TRIzol reagent (Invitrogen, USA) and treated with RNase-free DNase I (Takara Biotechnology, China). RNA integrity was assessed using the 2100 Bioanalyzer (Agilent). RNA integrity number (RIN) values were greater than 8 for all samples. Magnetic beads coated with oligo (dT) were used to isolate Poly (A)-containing mRNA. After synthesis of the first-strand cDNA using reverse transcriptase and random hexamer primers, the second strand of cDNA was synthesized using DNA polymerase I and RNaseH. Double-stranded cDNAs were subjected to end-repair using T_4_ DNA polymerase, Klenow fragment, and T_4_ polynucleotide kinase. The cDNAs were ligated with an adapter or an index adapter using T_4_ quick DNA ligase. The suitable adaptor-ligated fragments were selected for PCR amplification as templates. PCR was performed to enrich and amplify the selected fragments. The Agilent 2100 Bioanalyzer and ABI StepOnePlus Real-Time PCR System were used to quantify and assess the quality of the sample library. The cDNA library products were sequenced on the Illumina HiSeq^TM^ 2000 platform [The Beijing Genomics Institute (BGI), Shenzhen, China]. The transcriptome datasets are available at the NCBI Sequence Read Archive (SRA), under accession number SRP064996.

### Data Processing and *De novo* Assembly

Raw data or reads generated by Illumina platform were cleaned by removing adapter sequences, empty reads, and low-quality sequences (reads with ambiguous bases ‘N’). The clean reads were compared against the genome of ERM fungi (*Oidiodendron maius*^1^) using SOAP2 (Li et al., 2009). Any overlapping reads were discarded prior to performing assembly to ensure that all reads in the dataset were plant origin. Transcriptome *de novo* assembly was subsequently carried out using the short reads assembling program-Trinity (Grabherr et al., 2011), i.e., reads with overlaps were assembled to generate contigs, which were joined into scaffolds that were further assembled through gap filling to generate sequences called unigenes. Assembled unigenes from JZ and WJZ were taken into further process of sequence splicing, and redundancy was removed with sequence-clustering software to acquire non-redundant unigenes.

### Transcriptome Annotation

Unigenes were aligned to several protein databases using BLASTx (*E*-value < 10^-5^), including the NCBI non-redundant protein (Nr), Swiss-Prot (European Protein) database, the Kyoto Encyclopedia of Genes and Genomes (KEGG) pathway (Kanehisa et al., 2008), and the Cluster of Orthologous Groups of proteins (COG)^2^ databases. Sequence directionality was assigned according to the best alignments. When the different databases gave different results, the following priority structure was used to choose one unigene: NCBI Nr, Swiss-Prot, KEGG, and COG. When a unigene failed to align to any of the four databases, ESTScan (Iseli et al., 1999) was used to predict its coding regions and ascertain its sequence direction.

### Differentially Expressed Unigenes

To determine transcript abundance levels of unigenes, the uniquely mapped reads for a specific transcript were counted by mapping reads to assembled sequences using SOAP2 (Li et al., 2009). The RPKM (reads per Kb per million reads) values were calculated using Cufflinks program (Mortazavi et al., 2008; Trapnell et al., 2010). DEGs between the JZ and WJZ samples were obtained from RPKM values using a method modified by Audic and Claverie (1997). Fold changes for each unigene were calculated as the ratio of RPKM values. If the value of either JZ-RPKM or WJZ-RPKM was zero, 0.001 was used instead of 0 to measure the fold change. The significance of differential transcript abundance was computed using the FDR (False Discovery Rate) control method (Benjamini and Yekutieli, 2001) to justify the *p*-value, and only unigenes with an absolute fold change ≥2 and a FDR significance score ≤0.001 were used for subsequent steps of the analysis.

### Gene Ontology Enrichment and DEGs Pathway Analyses

Gene ontology (GO) term annotation (molecular function, biological process, and cellular component) was analyzed using the Blast2go software (version 3.0) (Ashburner et al., 2000; Conesa et al., 2005) based on Nr database. After obtaining GO annotation for each unigene, WEGO software (Ye et al., 2006) was used to perform GO functional classification for all unigenes and to understand the distribution of gene functions at the macro level. The analysis first maps all DEGs to GO terms in the database by virtue of calculating gene numbers for every term, followed by an ultra-geometric test to find significantly enriched GO terms in DEGs compared to the transcriptome background. Calculated *p*-values were subjected to a Bonferroni Correction with a corrected *q*-value ≤0.05 as a threshold. GO terms fulfilling this condition were defined as significantly enriched DEGs. This analysis recognized the main biological functions of identified DEGs. GO functional enrichment analysis also integrated the clustering analysis of expression patterns of DEGs. Additionally, the Blastall program was used to annotate the pathways of DEGs against the KEGG database (Kanehisa et al., 2008).

### qRT-PCR Analysis of Selected DEGs

In order to verify some differential expressed genes, a fourth plant growth experiment was performed. Seedlings of *R. fortunei* were grown in 480 culture vessels of which 240 were inoculated with Om19 (JZ) and the remaining were uninoculated as control (WJZ). The experiment was a randomized complete block design with three blocks, and each treatment had 80 vessels per block. Twenty vessels per treatment were randomly selected from each block on weeks 1, 3, 6, and 8. Roots of JZ and WJZ from each block were, respectively, collected, and total RNAs were extracted using TRIzol reagent (Invitrogen, USA). qRT-PCR was carried out to analyze expression levels of 11 DEGs, which are homologous to *SymRK. NORK. CCaMK. DM1. NRT1-1* and *NRT1-2. AMT3. GS-1* and *GS-2* (glutamine synthetase), *GOGAT-1* and *GOGAT-2* (glutamate synthase). The sequence identity of the 11 DEGs to other plant species ranged from 69 to 86 % (**Supplementary Table S1**). Gene specific primers were designed according to the cDNAs with Primer Premier software (version 5.0) (**Supplementary Table S2**). EF1α, previously tested as the most stably expressed gene, was used as an internal control. The first strand cDNA was synthesized using the PrimeScript II the first strand cDNA Synthesis Kit (Takara, Dalian, China). qRT-PCR was performed in a 20 μL reaction mixture containing 2x SYBR Master Premix Ex Taq II 12.5 μL (Takara, Dalian, China), 1 μL of cDNA template (1:5 dilution), and 1 μL of each corresponding primer for the gene of interest and EF1α. qRT-PCR of three biological replicates (from three blocks) for each treatment at four sampling periods was performed for 5 s at 95°C, 10 s at 56°C, and 20 s at 72°C using a LightCycler 480 II System. The relative expression levels were normalized and calibrated according to the 2^-ΔΔCT^ method (Schmittgen and Livak, 2008). For a given gene, the relative expression level was expressed as mean ± SE of three replicates.

## Results

### Seedling Growth and Om19 Colonization

**FIGURE 1:**
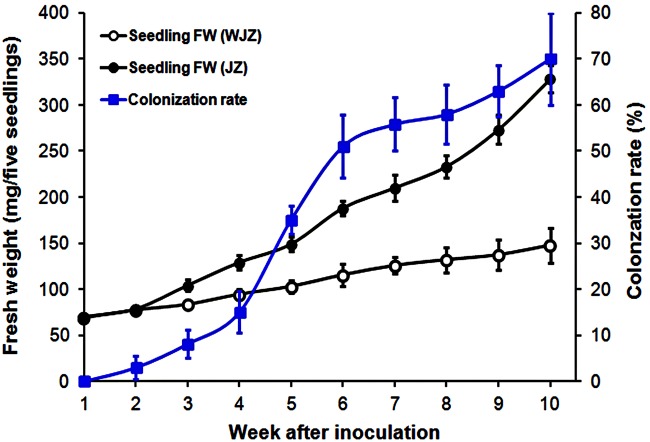
**Fresh weight (mg) of five *Rhododendron fortunei* seedlings inoculated (JZ) and uninoculated (WJZ) with an ericoid mycorrhizal (ERM) fungus (*Oidiodendron maius* var. maius Om19) as well as colonization rate of seedlings by the fungus during a 10-week growth period.** Bars represent standard errors (*n* = 6).

Seedlings grew healthily in the established system. Fresh weights of both WJZ and JZ seedlings continuously increased during the 10-week growth period (**Figure 1**). Starting from week 3, fresh weight of JZ seedlings increased significantly greater than those of WJZ. The significant growth increase corresponded to Om19 colonization of seedlings. The colonization rate in week 3 was 8% and increased to 70% in week 10 (**Figure 1**). Om19-colonized seedlings at the end of 10-week growth period had a mean root number of 8.2 and root length of 25.5 mm compared to 4.7 and 13.6 mm of the control seedlings. The mean leaf number and shoot height of Om19-colonized seedlings were 12.4 and 23.9 mm compared to 11.2 and 12.8 mm of control seedlings.

**FIGURE 2:**
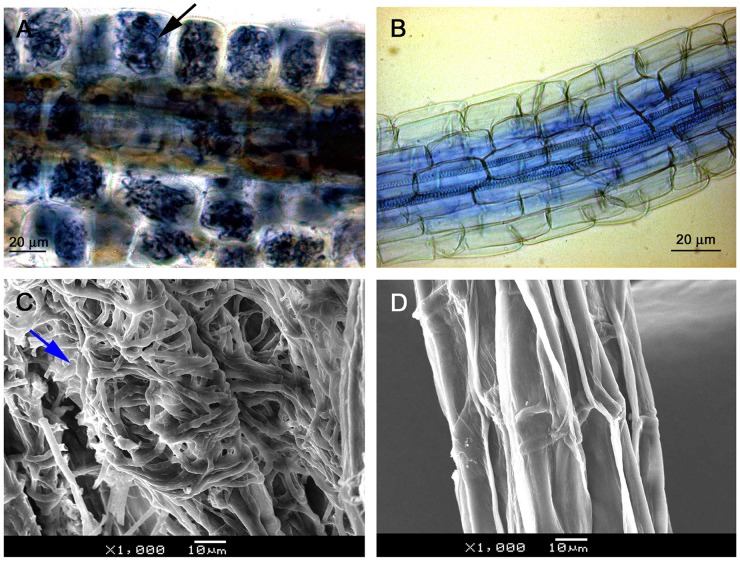
**Microscopic observation of *Rhododendron fortunei* roots infected by an ERM fungus (*Oidiodendron maius* var. maius strain Om19).** Root epidermal and cortical cells were completely filled with mycelium in week 6 as indicated by arrow **(A)**; no mycelium on root surface of seedlings **(B)**; SEM observation showing mycelia heavily surrounded roots (arrow) of seedlings inoculated with the fungus **(C)**; and the surface of control roots was clear **(D)**.

Light microscopy observation showed that mycelium was present on root surfaces of JZ seedlings as early as week 1. Fungal entry points on the epidermal cells were observed in week 2; the intracellular hyphal growth was observed in epidermal cells in week 3 and became clearly present in week 4. Root epidermal and cortical cells completely filled with mycelium in week 6 (**Figure 2A**). There was no mycelium on any root surface of control (WJZ) seedlings (**Figure 2B**). SEM observation also showed that mycelium heavily surrounded roots of JZ seedlings (**Figure 2C**) but the surfaces of control roots were clear (**Figure 2D**).

**FIGURE 3:**
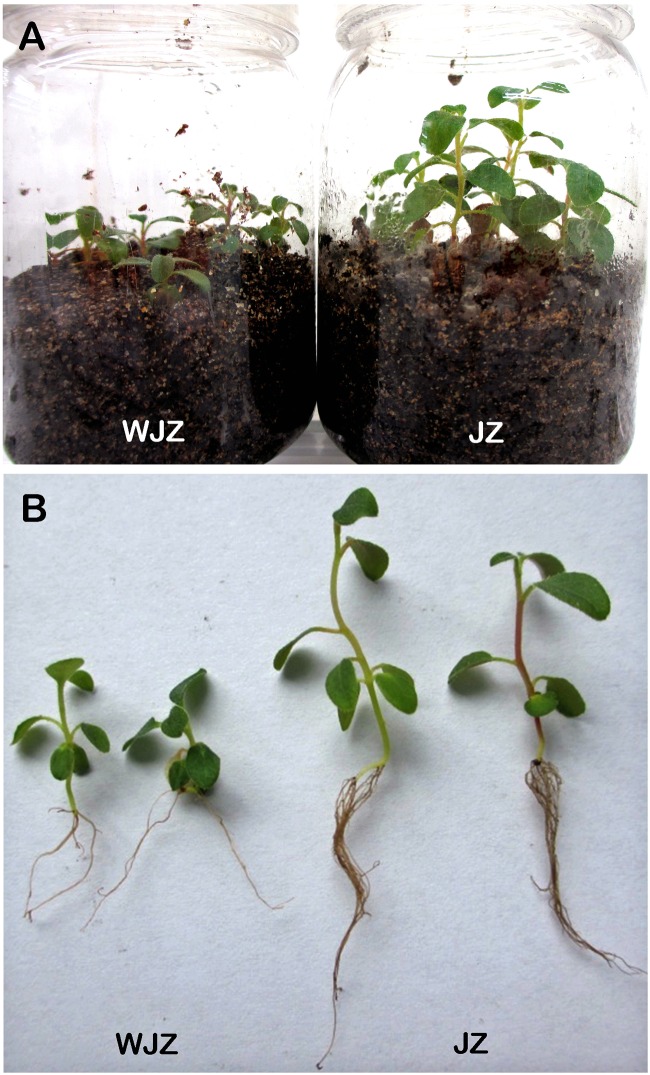
**Seedling of *Rhododendron fortunei* grown in a peat-based substrate inoculated (JZ) and uninoculated (WJZ) with a mycorrhizal fungus (*Oidiodendron maius* var. maius strain Om19) for 6 weeks **(A)** and representative seedlings after washing away substrate **(B)****.

**FIGURE 4:**
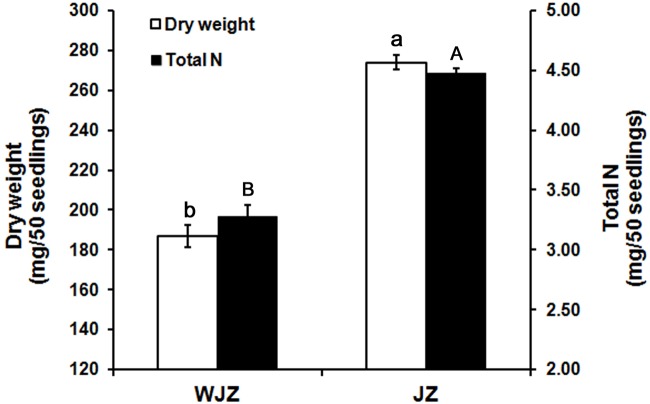
**Dry weight (mg) of 50 *Rhododendron fortunei* seedlings 6 weeks after inoculation (JZ) and uninoculation (WJZ) with an ERM fungus (*Oidiodendron maius* var. maius strain Om19) and total N in the 50 seedlings, respectively, where a and b indicate significant dry weight differences and A and B indicate significant total N differences between JZ and WJZ at *P* < 0.01 level based on Fisher’s protected least significant difference.** Bars represent standard errors (*n* = 6).

The second experiment, designed to study N uptake and biomass accumulation, showed that Om19-colonized seedlings (JZ) were much larger with more roots than those of control (WJZ) during a 6-week growth period (**Figure 3**). Total dry weight (root and shoot) of 50 JZ seedlings was 274 mg compared to 187 mg of 50 WJZ seedlings (**Figure 4**), a 46.6% increase. Total N in 50 JZ seedlings was 4.48 mg compared to 3.28 mg of 50 WJZ seedlings, a 36.6% increase.

### Transcriptome Sequencing Output and Assembly

After stringent quality checking and data cleaning, the Illumina sequencing platform generated 51.7 million, 90-bp long reads comprising 4.66 Gb nucleotides from WJZ and 50.6 million reads consisting of 4.56 Gb nucleotides from JZ (**Table 1**). The N percentage was 0.01% for both JZ and WJZ, suggesting that the proportion of unknown nucleotides in reads were extremely low. The sequencing quality was high since Q20 scores for reads of JZ and WJZ were 97.30 and 97.27%, respectively. The Q scores are defined as a property that is logarithmically related to the base calling error probabilities, and the Q20 means 1 error per 100 sequenced bases (Ewing et al., 1998). The reads were *de novo* assembled using the Trinity into 115,917 contigs with a N50 of 428 bp (50% of the assembled contigs had 428 bp or longer) for WJZ and 151,974 contigs with a N50 of 346 bp for JZ. The contigs were further assembled into scaffolds with paired-end read joining and gap-filling. Scaffolds were then assembled into 68,627 unigenes with a mean length of 458 bp for WJZ and 87,692 unigenes with a mean length of 410 bp for JZ (**Table 1**). Using the same strategy, a total of 70,720 unigenes with a mean length of 570 bp were obtained from both WJZ and JZ unigenes, which were called as all unigenes (**Table 1**).

**Table 1:** Summary of the sequence assembly after Illumina sequencing of *Rhododendron fortunei* roots uninoculated (WJZ) and inoculated (JZ) with an ericoid mycorrhizal fungus (*Oidiodendron maius* var. Maius Om19).

	WJZ	JZ	WJZ & JZ
Total clean nucleotides (bp)	4,657,028,220	4,557,459,600	
Total clean reads	51,744,758	50,638,440	
GC percentage	50.43%	49.47%	
Total number of contigs	115,917	151,974	
Mean length of contigs (bp)	291	265	
Total number of unigenes	68,627	87,692	
Mean length of unigenes (bp)	458	410	
The number of all-unigenes			70,720
Mean length of all-unigenes (bp)			570


### Functional Annotation of all Unigenes

For validation and annotation of the assembled unigenes, all 70,720 unigenes were searched against six databases using Basic Local Alignment Search Tool (BLASTX) with an *E*-value < 10^-5^, which resulted in 45,058, 36,399, 28,048, 24,986, 15,105, and 35,283 unigenes that were annotated to Nr, NT, Swiss-Prot, KEGG, COG, and GO databases, respectively. As a result, a total of 46,959 (66.4%) all unigenes were annotated to one or more of the databases. The *E*-value distribution of the top hits in the Nr database revealed that 45% of the mapped sequences showed significant homology (less than 1.0*E* - 45) (**Supplementary Figure S1A**), and 69.3 and 28.2% of the sequences had similarities greater than 60 and 80%, respectively (**Supplementary Figure S1B**). Of which 49.3% of the unigenes were homologous with sequences of *Vitis vinifera*; 12.7, 12.2, and 6.4% of the unigenes had a significant similarity with the sequences of *Ricinus communis. Populus trichocarpa*, and *Glycine max*, respectively (**Supplementary Figure S1C**).

The GO database comprises three ontologies: molecular function, cellular components, and biological processes. The basic units of GO are the “GO terms,” each belongs to a type of ontology. A total of 35,283 unigenes were assigned to 57 GO terms consisting of three domains: biological process, cellular component, and molecular function (**Supplementary Figure S2**). Some of the most common processes included cellular process, cell, cell part, and organelle. In contrast, only four genes were assigned to metallochaperone activity, and one gene was assigned to virion.

**FIGURE 5:**
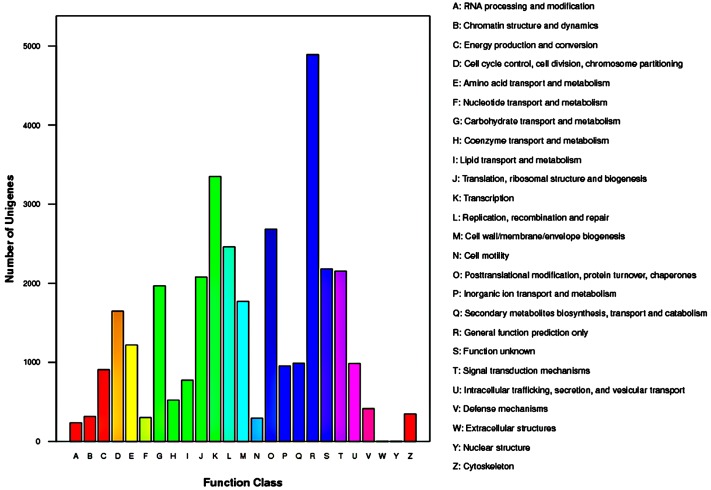
**Histogram representation of clusters of orthologous groups (COG) classification of *Rhododendron fortunei* transcriptome where all unigenes were assigned to 25 categories in the COG classification**.

The COG database contains classifications of orthologous gene products. The 15,105 unigenes which were annotated to COG database were distributed to 25 COG categories (**Figure 5**). Among the 25 COG categories, general function prediction represented the largest group (4,891 unigenes, accounting for 32.38%), followed by transcription (3,351, 22.18%), and post-translational modification/protein turnover/chaperones (2685, 17.78%). The smallest groups were nuclear structure (4, 0.03%) and extracellular structures (5, 0.03%).

To identify the biological pathways, 70,720 all unigenes were mapped to KEGG pathways, of which 24,986 were annotated to KEGG, and assigned to the 128 KEGG pathways. The most highly represented categories were metabolism pathway (6,241 unigenes), biosynthesis of secondary metabolites (2,486 unigenes), and endocytosis pathway (1,565 unigenes). However, only six unigenes were assigned to biotin metabolism pathway and four to betalain biosynthesis pathway. Additionally, a number of unigenes were only annotated to a single pathway.

### Functional Annotation of DEGs

**FIGURE 6:**
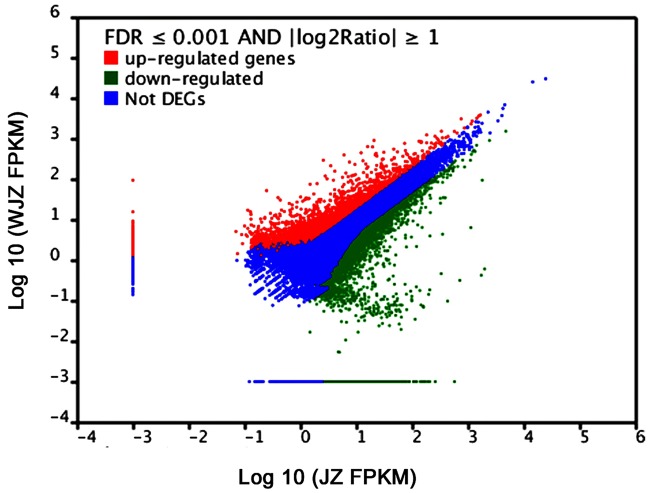
**FPKM distribution of differentially expressed genes (DEGs) from roots of *Rhododendron fortunei* inoculated (JZ) and uninoculated (WJZ) with an ERM fungus (*Oidiodendron maius* var. maius strain Om19) where upregulated DEGs are in red, downregulated DEGs are in green, and genes that were not differentially expressed in blue**.

A total of 48,148 unigenes were upregulated and 22,541 were downregulated due to the Om19 colonization using the Cufflinks (Trapnell et al., 2010). According to the threshold of the false discovery rate (FDR ≤ 0.001) and the absolute value of log2Ratio (≥1), 16,892 unigenes were found to be significantly differentially expressed. Of which 14,364 were upregulated and 2,528 were downregulated. The number of upregulated genes was over fivefold more than that of downregulated genes. A scatter plot showed a positive relationship of unigenes between JZ and WJZ (**Figure 6**), suggesting that the expression of most of the genes had a similar pattern and that a sizeable portion of the genes were differentially expressed due to the Om19 colonization.

**FIGURE 7:**
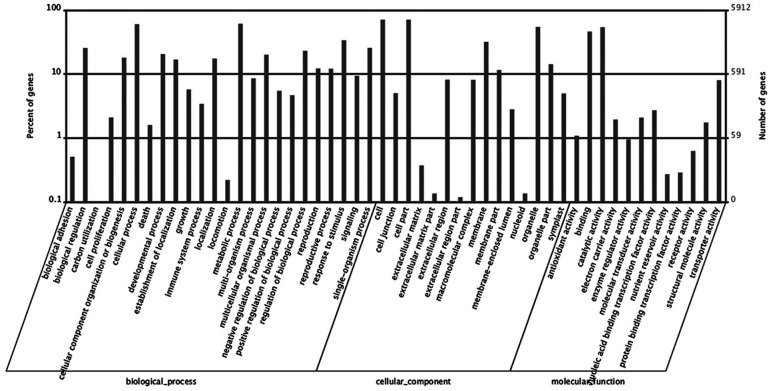
**Histogram of differentially expressed genes (DEGs) in roots of *Rhododendron fortunei* inoculated (JZ) and uninoculated (WJZ) with an ERM fungus (*Oidiodendron maius* var. maius strain Om19) which were classified based on gene ontology (GO)**.

Based on GO analysis, 5,939 of DEGs were further classified. They were categorized into 51 GO terms consisting of three domains: biological processes (24 terms), cellular components (15 terms), and molecular function (12 terms) (**Figure 7**). Within the biological processes catgory, cellular process, metabolic process, and response to stimulus were highly represented. In the cellular component category, cell and cell part dominated. In the molecular function group, observed binding and catalytic activity prevailed.

Kyoto Encyclopedia of Genes and Genomes is a large publicly available database for identifying enriched genes that may be part of metabolic or signal transduction pathways (Kanehisa et al., 2008). To explore the biological function of those differentially expressed genes, 4,620 DEGs were mapped to 267 pathways in the KEGG database. These genes were enriched in several important pathways, including metabolic pathways (1,398, 30.37%), biosynthesis of secondary metabolites (696, 13.4%), microbial metabolism in diverse environments (244, 5.29%), plant and pathogen interaction (237, 5.14%), plant hormone signal transduction (233, 4.83%), and starch and sucrose metabolism (193, 4.18%).

A series of unigenes related to symbiotic processes were upregulated in Om19-colonized roots. For example, unigene38932, a homology to a gene for 1-deoxy-D-xylulose 5-phopate synthase (DXS, EC2.2.1.7) and a key enzyme in methyl-D-erythrito 4-phosphate (MEP) pathway, was upregulated by a log-fold change of 4.0. More than 10 unigenes encoding to ATP binding cassette (ABC) transporters were upregulated by log-fold changes from 3 to 13. Unigene29489, a homologous of GRAS-type transcription factors in legumes was also upregulated in a log-fold change of 3.4. A total of 13 unigenes homologous to early nodulin proteins (ENOD) were identified and 7 of them were upregulated by log-fold changes greater than 10. Unigene1085, the common SYM genes *DMI1. DMI2*, and *DMI3* of *Medicago truncatula* (Genre et al., 2005) were also upregulated by log-fold change more than 2.6. Unigene24249, a homologous to lysine-motif (LysM) receptor kinase was upregulated by a log-fold change of 1.7. A group of unigenes related to CCaMK were upregulated by log-fold changes up to 13.7. Unigene37601, Nod factor binding lectin-nucleotide phosphohydrolase and unigene22160_All, NORK were upregulated by log-fold changes of 11.8 and 2.1, respectively. A total of 11 unigenes related to nuclear pore complex proteins (NUP) were also upregulated by log-fold changes from 11.5 to 15.6. Additionally, 251 DEGs were annotated as plant–pathogen interaction, of which 196 were upregulated by log-fold changes up to 15.2.

Unigenes involved in nitrogen metabolism were upregulated by log-fold changes from 1 to 16. Unigenes homologous to *NRT* were upregulated by log-fold changes from 1 to 13.5. Unigenes homologous to *AMT* family were also upregulated by log-fold changes from 1.2 to 13.9 (**Supplementary Table S3**), nitrate reductase and nitrite reductase upregulated by long-fold change of 2.2 and 11.4, respectively. *GS* and *GOGAT* were upregulated by log-fold change of 3 and 16.1, respectively.

The present study also identified a number of genes involving endocytosis (411, 8.91%), Fc-gamma R-mediated phagocytosis (395, 8.56%), glycerophospholipid metabolism (390, 8.45%), Gonadotropin-releasing hormone (GnRH) signal pathway (379, 8.22%), and ether lipid metabolism (365, 7.91%). Among them, 287 unigenes homologous to phospholipase D (PLD) (E.C. 3.1.4.4) were upregulated, of which 87 were up by long-fold changes greater than 10. PLD occurs in endocytosis, ether lipid metabolism, Fc-gamma R-mediated phagocytosis, glycerophospholipid metabolism, and GnRH signal pathways. Nine unigenes were homologous to extracellular-signal-regulated kinases (ERK1/2), and 7 of them were upregulated by more than 10-fold. ERK1/2 occurs in both Fc-gamma R-mediated phagocytosis and GnRH signal pathways.

**FIGURE 8:**
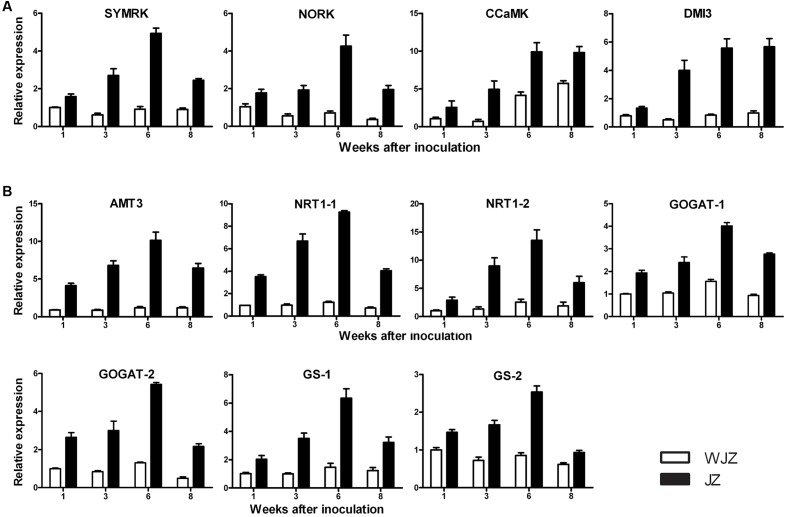
**qRT-PCR analysis of 11 selected differentially expressed genes in roots of *Rhododendron fortune* inoculated (JZ) and uninoculated (WJZ) with an ERM fungus (*Oidiodendron maius* var. maius strain Om19).** The relative expression levels were normalized and calibrated according to the 2^-ΔΔCT^ method. For a given gene, the relative expression level was expressed as mean ±SE (*n* = 3). **(A)** Genes related to symbiosis (*SymRK. NORK. CCaMK*, and *DM1*), **(B)** Genes related to N uptake and metabolism (*AMT3, NRT-1-1*, *NRT1-2. GOGAT-1. GOGAT-2. GS-1*, and *GS-2*).

qRT-PCR analysis showed that the selected 11 DEGs were highly upregulated in JZ roots ranging from 1.5 to 9.5 fold compared to their expressions in WJZ roots (**Figure 8**). The relative expression of DEGs homologous to *SymRK. NORK. CCaMK*, and *DMI3* continuously increased from weeks 1 to 6 in JZ roots; *SymRK* and *NORK* decreased in week 8; and *CCaMK*, and *DMI3* reached a plateau on week 8 (**Figure 8A**). The expression of these genes in WJZ roots varied to a limited extent over the 8-week period except for *CCaMK* that increased from week 6 to 8. DEGs homologous to N uptake (*AMT3. NRT1-1*, and *NRT1-2*) also continuously increased from weeks 1 to 6, and decreased in week 8 (**Figure 8B**). Correspondingly, *GOGAT-1. GOGAT-2. GS-1*, and *GS-2* showed the same expression pattern as *AMT3, NRT1-1*, and *NRT1-2*. Whereas the expression *AMT3. NRT1-1*, and *NRT1-2* as well as *GOGAT-1. GOGAT-2*, and *GS-1* in WJZ roots slightly increased.

## Discussion

The present study devised a simple system for establishing the symbiotic relationship between *R. fortunei* seedlings and an ERM fungus (*O. maius*). Om19 infection and colonization can be examined, and plant growth and N uptake can be quantified. Based on the system, high-quality RNA can be extracted from plant samples. Using the Illumina HiSeq^TM^ 2000, we were able to obtain high-quality transcript sequences as indicated by low proportion of undefined nucleotides (Ns) and low base-calling error probability. Our study is the first to document such a large number of genes involved in the symbiosis between an ERM fungus and *R. fortunei* and the genetic underpinning of mycorrhizal-mediated increase in N uptake and host plant growth.

### Om19 Colonization and Plant Growth

Since the peat-based substrate was autoclaved, and inoculation was performed under sterile conditions, the inoculated Om19 should be the only microbe in the system. The observed microbial infection and plant growth differences should be solely attributed to the interaction between the Om19 and *R. fortunei*. The colonization data suggested that by week 6, *R. fortunei* roots were thoroughly infected by Om19 (**Figure 1**). Om19 colonization enhanced root growth as greater root numbers and longer root lengths were observed in seedlings colonized by Om19 than the control (**Figure 3**). Large root systems result in a large root surface and shorter average half distance between root axes in the substrate for effective capture of nutrient elements (Chen and Gabelman, 2000). As a result, total N absorbed by Om19-colonized plants was higher than the control plants (**Figure 4**). Increased N uptake promoted plant growth, thus the dry weight of seedlings inoculated with Om19 was significantly greater than the control seedlings (**Figure 4**).

### Genes Related to Symbiosis

RNA-Seq identified 16,892 genes that were significantly differentially expressed in Om19-colonized roots. The highly expressed genes such as DXS in MEP pathway could result in the production of strigolactones. Strigolactones may be able to induce hyphal growth and branching of Om19. ABC transporters, particularly G-subfamily ones are thought to be putative strigolactone export component in *Petunia* (Kretzschmar et al., 2012). Unigenes homologous to ABC transporters were highly expressed in colonized roots of *R. fortunei*. GRAS-type transcription factors which were implicated in the regulation of strigolactone biosynthesis in legumes (Gutjahr and Parniske, 2013) were upregulated in *R. fortunei*. Subsequently, *ENOD* and the common *SYM* genes *DMI1. DMI2*, and *DMI3* were found to be significantly upregulated. qRT-PCR analysis also showed that *SYMRK. NORK. CCaMK*, and *DMI3* increasingly expressed from weeks 1 to 6 (**Figure 8A**). These genes are required for PPA induction in *Medicago truncatula* (Genre et al., 2005), and *DMI3* is required for a subset of genes to be induced during PPA formation (Siciliano et al., 2007). A gene homologous to LysM receptor kinase which is involved in symbiotic signal perception at the root plasma membrane (Broghammer et al., 2012) was increasingly expressed. These results suggest that the interaction between Om19 and *R. fortunei* probably occurred in weeks 1 to 3, and the symbiosis was probably well established before week 6. The significantly increased expression of *CCaMKs. DMI1. DMI2*, and *DMI3. NORK*, and *NUP* during the early stage of Om19 colonization of *R. fortunei* may suggest that the ERM fungus Om19 has similar processes as AMs in symbiosis with its host plants.

This study also identified a number of genes involving endocytosis, Fc-gamma R-mediated phagocytosis, glycerophospholipid metabolism, GnRH signal pathway, and ether lipid metabolism, which were significantly differentially expressed during the symbiosis. Endocytosis, a new and exciting research area in plant biology (Robinson, 2015), could be an important process for plants interacting with ERM fungi. It might be possible that PPA formation is associated with endocytosis. Phagocytosis plays an essential role in host–defense mechanisms through the uptake and destruction of infectious pathogens in animals. After opsonization with antibodies (IgG), foreign extracellular materials are recognized by Fc gamma receptors. Cross-linking of Fc gamma receptors initiates a variety of signals mediated by tyrosine phosphorylation of multiple proteins, which lead through the actin cytoskeleton rearrangements and membrane remodeling to the formation of phagosomes (Indik et al., 1995). Phosphoglycerolipids are essential structural constituents of membranes and some also have important cell signaling roles (Janda et al., 2013). One of the most important signaling lipids in plants is phosphatidic acid (Zhang and Xiao, 2015), which can activate or inactivate protein kinases and/or protein phosphatases involved in hormone signaling. It can also activate NADPH oxidase leading to the production of reactive oxygen species. GnRH was identified in animals, whose secretion from the hypothalamus acts upon its receptor in the anterior pituitary to regulate the production and release of the gonadotropins, luteinizing hormone (LH), and follicle-stimulating hormone (FSH). The GnRH is coupled to Gq/11 proteins to activate phospholipase C which transmits its signal to diacylglycerol (DAG) and inositol 1, 4, 5-trisphosphate (IP3). DAG activates the intracellular protein kinase C (PKC) pathway and IP3 stimulates release of intracellular calcium (Conn, 1994). To the best of our knowledge, there has been no report about the involvement of these genes in mycorrhizal colonization. Confirming their existence and their roles in the symbiotic relationships requires further investigation.

The Om19 colonization also induced plant defense responses as 251 DEGs were annotated to plant–pathogen interactions. Since seedlings colonized by Om19 were healthy and larger than the control ones, the defense reactions induced by Om19 could be mild and temporary, which is similar to the colonization of AM fungi with their hosts (Parniske, 2008). The defense responses may also have little effect on mycorrhizal infection (Hause and Fester, 2005; El-Khallal, 2007) as colonization rates increased (**Figure 1**). However, exact mechanisms underlying the host plant defense responses to Om19 infection and whether the responses affects its infection is currently unclear.

### N Metabolism

Inorganic N in the soil is preferentially taken up by AM fungi as NO_3_^-^ or ammonium. Once the N is absorbed by the extra-radical mycelia, it is converted into arginine for transport into intra-radical mycelia (Gomez et al., 2009; Tian et al., 2010). The arginine is broken down through the urease cycle into ammonium for transport into the plant (Govindarajulu et al., 2005). The present study used NO_3_^-^ as N source. The total N was substantially higher in Om19-colonized seedlings (**Figure 4**). RNA-Seq analysis identified 11 DEGs that were homologous to AMTs, and their expressions in Om19 colonized roots were upregulated by log-fold-changes from 1.3 to 13.9 compared to the uninoculated control (**Supplementary Table S3**). qRT-PCR analysis of *AMT3* also showed that its expression highly increased in Om19-colonized roots (**Figure 8B**). These results may suggest that mycelia of Om19, similar to AM fungi, absorbed NO_3_^-^, converted it into arginine, and then released ammonium to plants. Increased ammonium in plants may trigger *GS* and *GOGAT* activities. This study identified three unigenes homologous to *GS*, and nine unigenes to *GOGAT*. qRT-PCR analysis showed that the expression of *GS-1* and *GS-2* almost linearly increased in Om19 colonized roots from weeks 1 to 6 after inoculation (**Figure 8B**). GS and GOGAT are key metabolic enzymes that synthesize glutamine and glutamate, leading to the entrance of organic nitrogen in cellular metabolic pathways such as the biosynthesis of amino acids, nucleic acids and complex polysaccharides.

In addition to the aforementioned fungi mediated N uptake, Om19-colonized roots could also directly absorb NO_3_^-^ since unigenes homologous to *NRT*s were significantly upregulated. *NTR*s have two families: *NRT1* and *NRT2*. Members of the *NRT1* family mainly regulate the LATS and members of the *NRT2* family regulate HATS only (Kumar et al., 2003). We identified eight upregulated DEGs homologous to the *NRT1* family. qRT-PCR analysis showed that *NRT-1-1* and *NRT1-2* were highly upregulated in Om19-colonized roots (**Figure 8B**). Recent studies showed that *NRT1.1* from *Arabidopsis* is actually a dual-affinity transporter regulating NO_3_^-^ uptake by changing its affinity for NO_3_^-^ depending on the availability of NO_3_^-^ in the soil (Tsay, 2014; Sun and Zheng, 2015). It is unknown at present if *NRT1-1* or *NRT1-2* in *R. fortunei* plays the same roles as *NRT1.1* in *Arabidopsis*. The increased expression of *NRT1-1* and *NRT1-2* does suggest that *NRT*s were active in Om19-colonized roots.

The present study demonstrates that *R. fortunei* can use NO_3_^-^ as an N source under acidic growing conditions. Seedlings grew healthy in the peat-based substrate with a pH of 5.2, and seedling biomass linearly increased over time (**Figure 1**). In addition to using inorganic N, ERM fungi are capable of enzymatically degrading organic N from substrates (Read, 1996; Yang and Goulart, 1997). The increased expression of *AMT3. NRT1-1*, and *NRT1-2* from weeks 1 to 6 may suggest that the colonized roots mainly absorbed available NO_3_^-^ during this growth period. Since no additional nutrients were provided after transplanting, the decreased expression of these genes in week 8 makes us speculate that Om19 might start to degrade the peat substrate, and seedlings might begin to take up organic N. Recent study showed that *O. maius* symbionent expressed a full complement of plant cell wall-degrading enzymes in symbiosis, suggesting its saprotrophic ability in sphagnum peat (Kohler et al., 2015). At this point, whether Om19 enzymatically degraded organic N from the peat requires further investigation.

Nevertheless, the total N in 50 seedlings colonized by Om19 was 36.59% greater than the control seedlings (**Figure 4**), suggesting that Om19-colonization contributed significantly to N absorption. The increased uptake is likely attributed to Om19-mediated bioavailability of N and direct NO_3_^-^ absorption. A total of 51 DEGs were identified which are related to the nitrogen metabolism, and most of the DEGs were dramatically upregulated in Om19 colonized roots. Due to the increased N uptake, many pathways including energy and nutrient metabolism, glycolysis/gluconeogenesis, pentose phosphate pathway, TCA cycle, plant hormone signal transduction, starch and sucrose metabolism, and amino sugar and nucleotide sugar metabolism became more active as most of the genes mapping to the above pathways were greatly upregulated in roots inoculated with Om19. The Om19-colonized seedlings are metabolically more active than the control seedlings. Thus, Om19-colonized seedlings grew significantly larger than control ones.

## Conclusion

Ericaceous shrubs such as *Calluna vulgaris. Rhododendron* spp., and *Vaccinium* spp. occur both in open heathland communities and in forest ecosystems as understory vegetation (Read, 1996). Soils in those habitats are typically low in available nutrients and plants grown in such soils are often stressed by different factors such as low pH, metal availability, water availability, and high or low temperatures (Read, 1996; Cairney and Meharg, 2003). Cortical cells of ericaceous plants never form root hairs as those in the other plant families. Thus, the ability to form symbiotic relationships with ERM fungi is consider to be critical to the success of ericaceous plants in such stressful soil conditions (Read, 1996; Cairney and Meharg, 2003). The present study documented that 16,892 genes were differentially expressed in an ERM fungus colonized roots. Such a large number of gene expressions may suggest that the long-history co-evolution between *R. fortunei* and ERM fungi has fine-tuned plant genetic networks that particularly fit the unique ecosystem where soils are poor and acidic in a mutually beneficial way.

## Author Contributions

DP and CZ conceived and designed the experiments. XW conducted the experiments, analyzed the data, and drafted the manuscript. JC participated in data analysis, wrote, and revised the manuscript. The final version was approved by all authors.

## Conflict of Interest Statement

The authors declare that the research was conducted in the absence of any commercial or financial relationships that could be construed as a potential conflict of interest.

The reviewer CF and handling Editor declared their shared affiliation, and the handling Editor states that the process nevertheless met the standards of a fair and objective review.

## Footnotes

1 http://genome.jgi-psf.org/Oidma1/Oidma1.home.html

2 http://www.ncbi.nlm.nih.gov/COG/
