# Supplementary material for: Differential Gene Expression in Rhododendron fortunei Roots Colonized by an Ericoid Mycorrhizal Fungus and Increased Nitrogen Absorption and Plant Growth
Source: Front Plant Sci. 2016 Oct 25;7:1594. doi: 10.3389/fpls.2016.01594 (PMC5078686; doi:10.3389/fpls.2016.01594)
Supplement: Supplementary file 1 [file Table_1.DOC]

Table S1. Selected 11 DEGs related to signal transduction in mycorrhizal symbiosis and nitrogen (N) uptake and metabolism and their sequence identities with known gene sequence in Genebank database.

1. DEGs related signal transduction

| Gene ID | Size  (bp) | Homologous protein | Organism origin | Accession Number | Query cover | E-value | Identity |
| --- | --- | --- | --- | --- | --- | --- | --- |
| SYMRK (Unigene22160_All) | 518 | Nodulation receptor kinase-like | *Vitis vinifera* | XM_002272019.3 | 52% | 4e-73 | 83% |
|  |  | Nodulation receptor kinase-like | *Populus euphratica* | XM_011013757.1 | 45% | 6e-71 | 86% |
|  |  | Nodulation receptor kinase-like | *Citrus sinensis* | XM_006466295.2 | 45% | 3e-68 | 85% |
| NORK(CL4176.Contig2_All) | 561 | Nodulation receptor kinase-like | *Populus euphratica* | XM_011013757.1 | 83% | 7e-64 | 72% |
|  |  | Nodulation receptor kinase-like | *Sesamum indicum* | XM_011101160.1 | 91% | 9e-63 | 71% |
|  |  | Nodulation receptor kinase-like | *Vitis vinifera* | XM_002272019.3 | 99% | 5e-53 | 70% |
| CCaMK (Unigene32009_All) | 343 | Calcium-dependent protein kinase 19 | *Vitis pseudoreticulata* | KR153947.1 | 37% | 3e-15 | 75% |
|  |  | Calcium and calcium/calmodulin-dependent serine/threonine-protein kinase-like | *Jatropha curcas* | XM_012229518.1 | 34% | 3e-15 | 77% |
|  |  | Calcium and calcium/calmodulin-dependent serine/threonine-protein kinase | *Vitis vinifera* | XM_010660713.1 | 37% | 3e-15 | 75% |
| DMI3 (Unigene3610_All) | 595 | Calcium and calcium/calmodulin-dependent serine/threonine-protein kinase | *Vitis vinifera* | XM_010660713.1 | 86% | 3e-158 | 84% |
|  |  | Calcium/calmodulin-dependent serine/threonine-protein kinase-like | *Prunus mume* | XM_008233660.2 | 84% | 7e-153 | 84% |
|  |  | Calcium/calmodulin-dependent serine/threonine-protein kinase | *Pyrus x bretschneideri* | XM_009340973.1 | 84% | 1e-143 | 83% |

1. DEGs related N uptake and metabolism

| Gene ID | Size  (bp) | Homologous protein | Organism origin | Accession Number | Query cover | E-value | Identity |
| --- | --- | --- | --- | --- | --- | --- | --- |
| AMT3 (CL4699.Contig1_All) | 1837 | Ammonium transporter 3 member 1-like | *Vitis vinifera* | XM_002272699.2 | 72% | 0.0 | 79% |
|  |  | Ammonium transporter 3.1-like protein | *Camellia sinensis* | KP338998.1 | 72% | 0.0 | 78% |
|  |  | Ammonium transporter 3 member 1-like | *Eucalyptus grandis* | XM_010027394.1 | 73% | 0.0 | 78% |
| NRT1-1 (Unigene35804_All) | 578 | NRT1/ PTR family 2.13-like | *Citrus sinensis* | XM_006483193.2 | 91% | 2e-70 | 72% |
|  |  | Nitrate transporter 1.7 | *Theobroma cacao* | XM_007044320.1 | 97% | 4e-67 | 71% |
|  |  | NRT1/ PTR family 2.13-like | *Eucalyptus grandis* | XM_010046396.1 | 99% | 9e-63 | 70% |
| NRT1-2 (Unigene17904_All) | 721 | NRT1/ PTR family Y 2.13 | *Vitis vinifera* | XM_002269304.2 | 73% | 3e-83 | 73% |
|  |  | NRT1/ PTR family 2.13 | *Pyrus x bretschneideri* | XM_009362133.1 | 74% | 3e-76 | 72% |
|  |  | NRT1/ PTR family 2.13-like | *Prunus mume* | XM_016792118.1 | 73% | 5e-74 | 72% |
| GS-1 ( CL10295.Contig2_All) | 840 | Glutamine synthetase precursor | *Juglans nigra* | AF169795.1 | 94% | 0.0 | 84% |
|  |  | Glutamine synthetase leaf isozyme | *Ricinus communis* | XM_002516755.2 | 93% | 0.0 | 84% |
|  |  | Glutamine synthetase leaf isozyme | *Jatropha curcas* | XM_012214101.1 | 92% | 0.0 | 83% |
| GS-2 (CL1213.Contig2_All) | 402 | Glutamine synthetase mRNA | *Camellia sinensis* | JQ925872.1 | 87% | 3e-111 | 86% |
|  |  | Nucifera protein fluG | *Nelumbo nucifera* | XM_010268979.1 | 81% | 6e-82 | 82% |
|  |  | Protein fluG | *Citrus sinensis* | XR_372458.2 | 87% | 4e-78 | 79% |
| GOGAT-1 (CL8980.Contig3_All) | 3126 | Glutamate synthase [NADH] | *Vitis vinifera* | XM_010664684.1 | 99% | 0.0 | 83% |
|  |  | Glutamate synthase [NADH] | *Jatropha curcas* | XM_012234693.1 | 99% | 0.0 | 81% |
|  |  | Glutamate synthase [NADH] | *Citrus sinensis* | XM_006490448.2 | 99% | 0.0 | 81% |
| GOGAT-2 (Unigene19965_All) | 939 | Glutamate synthase [NADH] | *Populus euphratica* | XM_011003602.1 | 68% | 0.0 | 83% |
|  |  | Glutamate synthase [NADH] | *Vitis vinifera* | XM_010664684.1 | 78% | 0.0 | 80% |
|  |  | Glutamate synthase [NADH] | *Ricinus communis* | XM_015715713.1 | 68% | 3e-179 | 82% |
