# Supplementary material for: Differential Gene Expression in Rhododendron fortunei Roots Colonized by an Ericoid Mycorrhizal Fungus and Increased Nitrogen Absorption and Plant Growth
Source: Front Plant Sci. 2016 Oct 25;7:1594. doi: 10.3389/fpls.2016.01594 (PMC5078686; doi:10.3389/fpls.2016.01594)
Supplement: Supplementary file 2 [file Table_2.DOC]

**Table S2 qRT-PCR primers**

| **Reference gene** | **Sequence** |
| --- | --- |
| EF1α-F | TGTCATCGATGCTCCTGGAC |
| EF1α-R | TCTCGGGTCTGACCACCCTT |
|  |  |
| **Selected DEGs** |  |
| SYMRK-F | AGTGGAGCTTGGTCGAATGG |
| SYMRK-R | AATGCATCCTCCAGCTCTCG |
|  |  |
| NORK-F | GACCGCTCCCCTCCAGTATG |
| NORK-R | CCCGTAAGATCATTGTGGCTCAG |
|  |  |
| CCaMK-F | GGGACGGGAAACAAGAACAC |
| CCaMK-R | AGTGTTTTGATGGCTACATGTTG |
|  |  |
| DMI3-F | ACAGAACCAGGGAAATTGGATGAG |
| DMI3-R | GCGAAGGGAGGAGAGGACTAC |
|  |  |
| GS-1-F | CTTTTGATGGCGTCCTTGAG |
| GS-1-R | TCCCACGGTTCCATAAGTGC |
|  |  |
| GS-2-F | GCCAACACGAGTTCCTGATGC |
| GS-2-R | CCATACAGCGAGCGGATTGC |
|  |  |
| GOGAT-1-F | GCATTGAGCAAGAGTACACCTTAC |
| GOGAT-1-R | TGATTCCAGCATACAAGCAAGCC |
|  |  |
| GOGAT-2-F | TGACTCCACACCCTACTGTTCTAC |
| GOGAT-2-R | ATGCGATTTCAAACTGACCTTTCC |
|  |  |
| NRT-1-F | AGTGTTGCCAATGCCCTATTCTTC |
| NRT-1-R | ATATCGTTTGTCAGCCAGTTCGG |
|  |  |
| NRT-2-F | AAGTTCAACATGGAGCAGGCATC |
| NRT-2-R | AGCGAAGGAGGCAACAACAATG |
|  |  |
| AMT-F | TCCTCCTCATTTCGTATATGTGGTAG |
| AMT-R | GCGTTGTCTCCTTTGTTCAACC |
