# Supplementary material for: Differential Gene Expression in Rhododendron fortunei Roots Colonized by an Ericoid Mycorrhizal Fungus and Increased Nitrogen Absorption and Plant Growth
Source: Front Plant Sci. 2016 Oct 25;7:1594. doi: 10.3389/fpls.2016.01594 (PMC5078686; doi:10.3389/fpls.2016.01594)
Supplement: Supplementary file 3 [file Table_3.DOC]

Table S3. Unigenes homologous to AMT (ammonium transporter) upregulated in roots of *Rhododendron fortunei* seedlings colonizedn by an ericoid mycorrhizal fungus Om19.

| GeneID (16846) | log2(JZ_FPKM/WJZ_FPKM) | Up/Down | p-value | FDR | Nr-annotation |
| --- | --- | --- | --- | --- | --- |
| Unigene26160_All | 13.8959 | Up | 2.07E-52 | 6.34E-51 | Uncharacterized protein LOC100191646 [*Zea mays*] |
| Unigene28491_All | 13.3674 | Up | 5.43E-19 | 6.19E-18 | Uncharacterized protein LOC100191646 [*Zea mays*] |
| Unigene33362_All | 13.2824 | Up | 1.54E-20 | 1.89E-19 | PREDICTED: ammonium transporter 3 member 1-like [*Vitis vinifera*] |
| Unigene36979_All | 12.3199 | Up | 5.43E-19 | 6.17E-18 | SORBIDRAFT_03g041140 [*Sorghum bicolor*] >gi|241928681|gb|EES01826.1| |
| Unigene37094_All | 12.2355 | Up | 6.75E-16 | 6.49E-15 | Uncharacterized protein LOC100191646 [*Zea mays*] |
| Unigene32989_All | 6.264 | Up | 4.86E-22 | 6.38E-21 | Uncharacterized protein LOC100191646 [*Zea mays*] |
| CL4699.Contig1_All | 2.0507 | Up | 9.66E-106 | 6.20E-104 | PREDICTED: ammonium transporter 3 member 1-like [*Vitis vinifera*] |
| Unigene3091_All | 1.994 | Up | 2.80E-26 | 4.32E-25 | PREDICTED: ammonium transporter 1 member 3 [*Vitis vinifera*] |
| CL4699.Contig2_All | 1.9623 | Up | 2.56E-17 | 2.68E-16 | Ammonium transporter, putative [*Ricinus communis*] >gi|223537874|gb|EEF39489.1| |
| Unigene11153_All | 1.4798 | Up | 1.12E-08 | 6.14E-08 | Ammonium transporter [*Populus trichocarpa*] >gi|222850514|gb|EEE88061.1| |
| Unigene24604_All | 1.2778 | Up | 1.62E-04 | 4.90E-04 | Unnamed protein product [*Vitis vinifera*] |
