# Supplementary figures and images for: Differential Gene Expression in Rhododendron fortunei Roots Colonized by an Ericoid Mycorrhizal Fungus and Increased Nitrogen Absorption and Plant Growth
Source: Front Plant Sci. 2016 Oct 25;7:1594. doi: 10.3389/fpls.2016.01594 (PMC5078686; doi:10.3389/fpls.2016.01594)

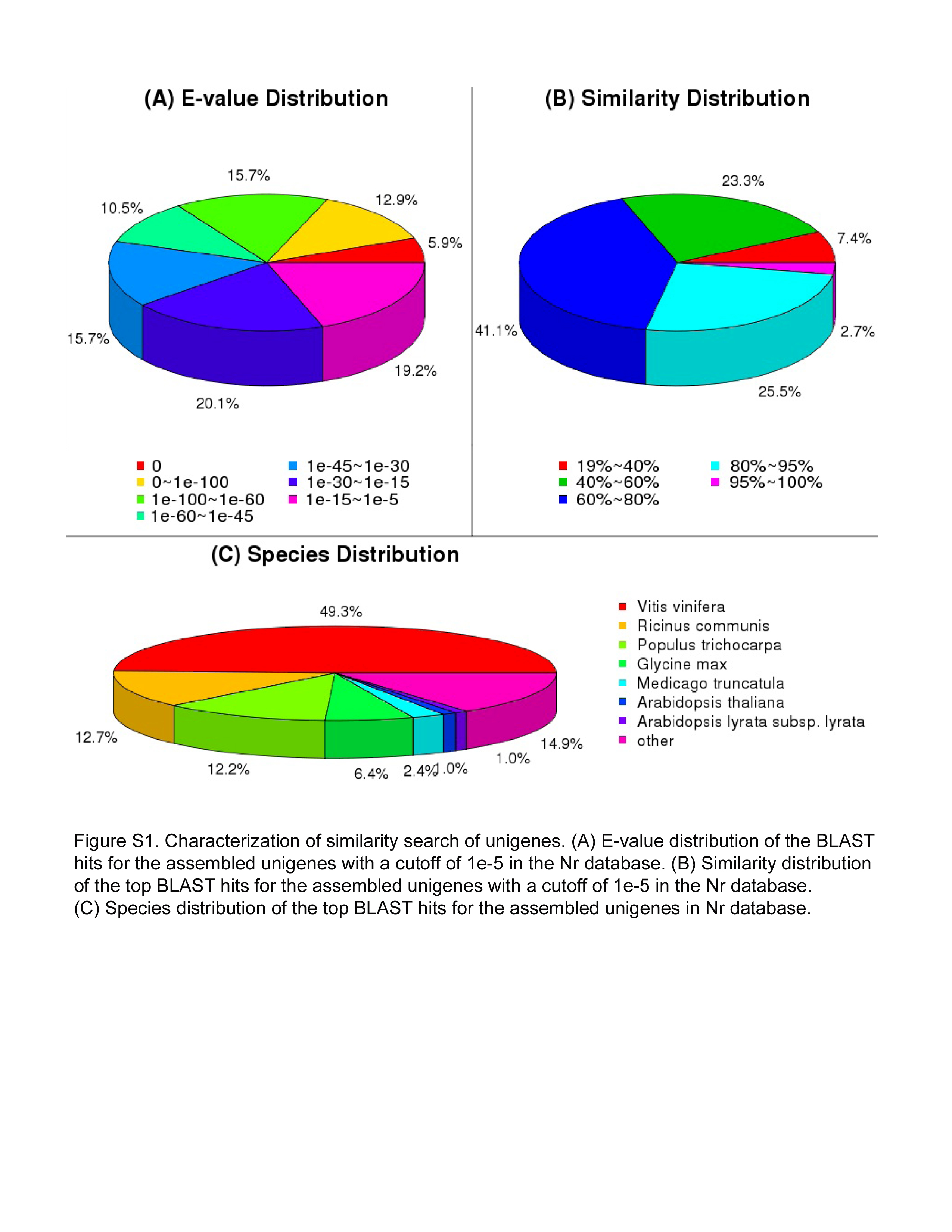

Supplement: Supplementary file 4 [file Image_1.JPEG]

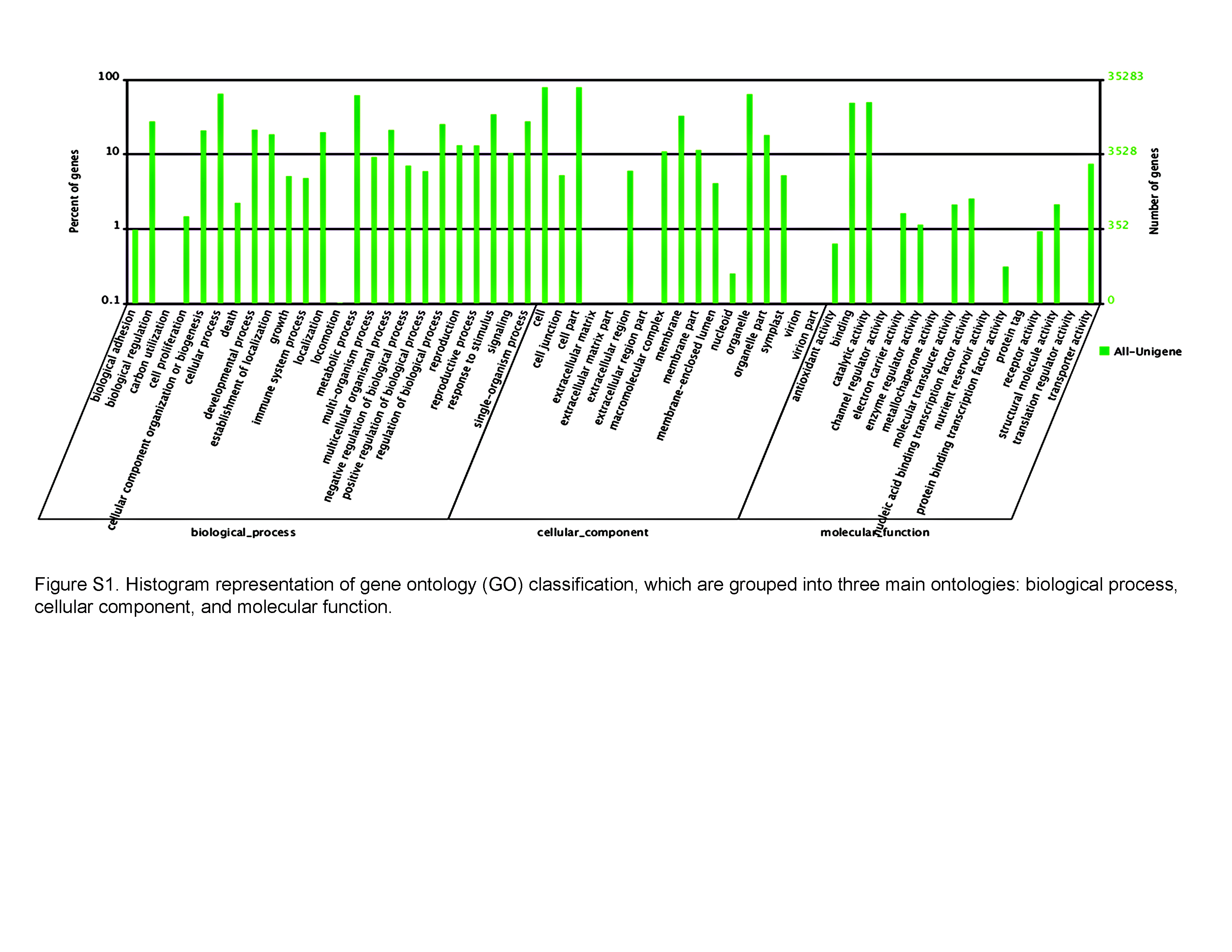

Supplement: Supplementary file 5 [file Image_2.JPEG]
